# Factors influencing maternal health choices among women of reproductive age in Hausa communities in Ibadan, Nigeria: an exploratory study

**DOI:** 10.11604/pamj.2021.38.90.12264

**Published:** 2021-01-27

**Authors:** Titilayo Dorothy Odetola, Ruth Ashadzi Salmanu

**Affiliations:** 1 Department of Nursing, University of Ibadan, Ibadan, Nigeria

**Keywords:** Family, planning, place, delivery, Hausa

## Abstract

**Introduction:**

decision making is a vital aspect of women's reproductive life. In an attempt to fulfil the desire to procreate, women's lives are lost especially in developing countries where medical care is still inadequate. The researchers sought to explore the Hausa people's culture as regards maternal health choices concerning modern family planning methods, delivery places and parity.

**Methods:**

a cross-sectional study using mixed methods was conducted among women of reproductive age (N=253) in three Hausa communities in Ibadan. Based on the objectives, semi-structured questionnaire and in-depth interview guide were used for data collection. Quantitative data were analysed using Chi-square and the level significance set at 0.05 while qualitative data were analysed thematically.

**Results:**

over fifty percent respondents had more than 3 children and about one third (33.9%) preferred having more than 4 children. Nearly all participants (94%) were aware of modern family planning methods but only 49.0% used them. The most widely used methods are injectables (22.0%) and condoms (20.6%). Almost half of the study population (46.5%) delivered their babies at home with assistance from other residents with most preferring home delivery for various reasons.

**Conclusion:**

although the level of awareness about family planning methods is quite satisfactory, however, the level of contraceptive uptake is still sub-optimal. Most Hausa women prefer home delivery which predisposes them to associated complications. Findings further identified various reasons for maternal health choices and provided insights on viable nursing interventions that can be adopted to promote skilled birth delivery to reduce maternal morbidity and mortality.

## Introduction

Population growth has been a problematic issue all over the world with maternal and infant mortality rates as well as malnutrition among children relatively high. This is as a result of inadequate resources and facilities to cater for this growing population. Family planning is a recognized basic human right that enables individuals and couples to determine the spacing and number of their children [1]. Attempts to control population increase started from the early men; therefore, birth control is as old as man himself. Before the introduction of modern methods, Africans had methods of fertility regulation. Nigerian culture includes many myths, rituals and the use of herbs in attempts to regulate women´s fertility. Although many of these traditional methods of family planning have no harmful effects on a woman´s health, some however, do have dangerous or counterproductive effects [2]. A woman´s ability to space and limit her pregnancies has a direct influence on her health and well-being as well as on the outcome of each pregnancy [3]. In effect, family planning provides opportunity for regulation and control of the rate of child birth by individuals, both married and unmarried. The National Demographic Health Survey (2008) revealed that 72 percent of all women and 90% of all men know at least one contraceptive method. Male condoms, the pill and injectables are the most widely known methods [4]. Most women prefer to align with the populist opinion that allows women to bear as many children as their husbands decide; and prefer simple modern methods like the use of condom. This, they believe, would not affect them negatively anytime they decide to return to fertility [2].

In developing nations like Nigeria, children are valued, as they not only demonstrate the masculinity of men but equally provide the extra useful hands on the farms in communities such as the Hausa settlements where agriculture is the major source of income. Besides, aged parents and extended family relations depend on their children for maintenance at old age [5]. Hence, they are reluctant to limit the number of children they give birth to [6]. Diverse socio-cultural beliefs are in line with strong preference for polygamy and these beliefs hold that children are a blessing from God and insurance against old age. The fact that a man has the final say in the family has also to a great extent determined what, when and probably how a family planning method would be used [3]. The Hausa community is patrilineal in nature, with a strong male influence on many decisions concerning the household, including reproduction. This makes the attitude of the male counterpart towards family planning and the preferred number of children important factors influencing fertility level among the Hausas [7]. Childbirth and its process is one of the most significant events in the life of a woman. The time of birth, as well as postpartum, are the most critical period in a woman´s life especially in the developing world. Hence, the choice of place of delivery for a pregnant woman is important to maternal health care [8]. Every day, approximately 1,000 women die globally from preventable causes related to pregnancy and childbirth, of which, 99% of all maternal deaths occur in developing countries [9].

Culture and religion, are so much more inculcated in the lives of the population than newly introduced reproductive freedom and women´s autonomy movements making the former very difficult to overcome. This is particularly true in those areas and societies such as the Hausa´s, where tradition remains important in everyday life [8]. According to Nigerian Demographic Health Survey report (2013), a significant proportion of mothers in developing countries deliver at home, with most births unattended by skilled personnel. About half of mothers in Nigeria received at least four antenatal visits while being pregnant. Only about 38% of births in Nigeria are delivered by a skilled health provider, such as a midwife, doctor or nurse. This was far lower in three of the northern states [10]. In diverse contexts, individual factors, including maternal age, parity, education and marital status; household factors including family size and household wealth; and community factors including socioeconomic status, community health infrastructure, region, rural/urban residence, available health facilities and distance to health facilities all determine place of delivery and these factors also interact in different ways [11].

The researchers therefore sought to deeply understand the Hausa culture, as regards decision-making about acceptance and preference for modern family planning methods, delivery places and preferred number of children. The findings from this study provided insight into how these communities could be assisted to reduce the rate of maternal morbidity and mortality. Heads of families and indeed women should be educated on the need for modern contraceptive uptake, improved uptake of skilled birth delivery and appropriate choice of delivery places to improve maternal and child outcomes.

## Methods

This is a cross-sectional mixed methods study which was conducted in three Hausa communities in Ibadan, namely; Ojo´o, Sasa and Sabo. The research sample was taken from these three communities through purposive sampling technique. A total of two hundred and fifty three (253) Hausa women were recruited in the quantitative aspect, based on the calculated sample size, while ten key persons in these communities (five men and five women) were purposively recruited for the qualitative aspect. Two research instruments (a semi-structured interviewer administered questionnaire and an in-depth interview guide), were developed by the researchers. The questionnaire contained six sections to explore participant´s demographic data; women´s awareness and use of family planning methods; factors influencing choice of family planning methods; impact of significant other´s decision on the acceptance of family planning; factors influencing family size and; factors influencing the choice of delivery place.

Each section of the questionnaire provided answer to a different research question as indicated above. The interview guide contained the introductory and question sections. Both instruments asked questions that were relevant to the topic and they were administered to the right group of participants, making them valid and reliable. The research hers obtained approval from the University of Ibadan/University College Hospital Ethical Review Committee after due process. Data collection was done through questionnaire administration and in-depth interviews of ten key people in Sasa community including the chiefs, security officers and women leaders, male and female politicians. The participants were visited in their homes after due process of community entry with the assistance of a guide. Interviews were made in Hausa language and tape recorded after obtaining consent from the participants. Thereafter, the recorded data were transcribed for theme and subtheme coding. Quantitative data were presented in percentages, frequencies, tables, bar and pie charts. Data analyses were carried out using Chi-square and Fisher exact test with the level of significance set at 0.05. Qualitative data were analysed thematically.

For the quantitative aspect, using the Fisher´s sample size calculation formula, with consideration for attrition, a study population of two hundred and fifty three (253) participants were involved in the study. The sample size was derived using the sample size formula below:

$$n=\frac{N}{1+N{\left(e\right)}^{2}}$$

(where: n=required sample size; N=accessible population (estimated number of Hausa women of the reproductive age in HAUSA communities in Ibadan, N=600) e=level of error tolerance (5%)=240 women). Adjusting the sample size for 5% non-response.

$$n_{f}=\frac{n}{1-Nr}$$

N_f_=252.6, calculated sample size with attrition = 253 participants.

## Results

**Figure 1:**
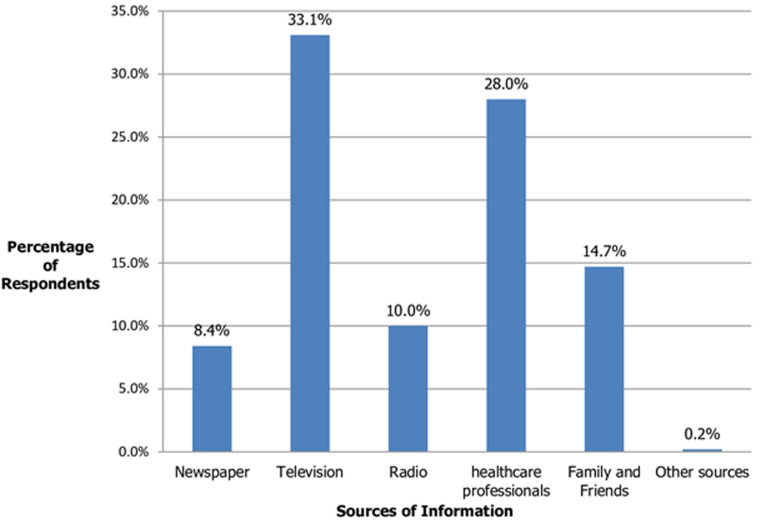
participants' sources of information about family planning

**Figure 2:**
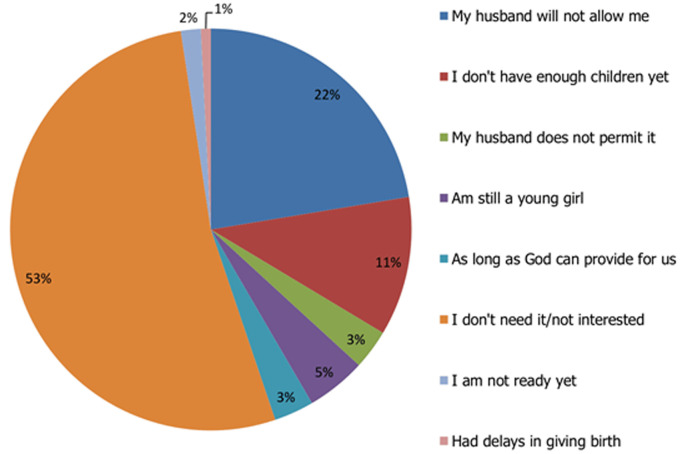
participants' reasons for not using modern family planning methods

As shown in Table 1, the mean age of participants was 30.6 ± 8.7 years; with the greater portion of participants falling between 26 and 35 years of age. Majority of the participants (95.5%) practised Islam. Thirty-four (13.8%) participants had no formal education while one hundred (40.8%) had primary education. Eighty three (33.9%) participants preferred to have more than 4 children; eighty one (33.1%) preferred to have 2 children or less while eighty one (33.1%) preferred to have between two and four children. With reference to how many children the participants preferred to have, one interviewee expressed her opinion thus: “*I have 8 children and myself and my husband planned to have a dozen children but he is deceased now so I have to stop here*” (IDI, women leader, Sasa). Two hundred and five (83.7%) participants had no monthly income. Two hundred and thirty one (94.3%) participants were aware of modern family planning methods with the major sources of information being television (33.1%) and healthcare workers (28.0%) (Figure 1). Only 43.3% of the participants were on family planning. Majority of the remaining participants, who do not use any family planning methods, gave reasons like not being interested and husbands´ disapproval (Figure 2). The most commonly used modern methods of contraception among the participants were injectables (22.0%), condoms (20.6%) and implants (18.4%).

**Table 1:** demographic characteristics of participants

Variable	Frequency	Percentage
**Age grouping (years)**		
15-25	84	34.3
26-35	88	35.9
>35	73	29.8
Mean ± SD		30. 6 ± 8.7
**Location**		
Sabo	120	49.0
Sasa	81	33.1
Ojo'o	44	18.0
**Marital status**		
Singled	4	1.6
Married	232	94.7
Separated	1	0.4
Divorced	3	1.2
Widowed	5	2.0
**Nationality**		
Nigerian	244	99.6
Ghanaian	1	0.4
State of origin		
Kastina	33	13.5
Kano	65	26.5
Bauchi	5	2.0
Jigawa	10	4.1
Kaduna	26	10.6
Nasarawa	13	5.3
Sokoto	36	14.7
Borno	23	9.4
Plateau	2	0.8
Kebbi	11	4.5
Gombe	5	2.0
Yobe	5	2.0
Zamfara	7	2.9
Niger	4	1.6
**Religion**		
Christianity	11	4.5
Islam	234	95.5
**Educational qualification**		
None	34	13.8
Primary	100	40.8
Secondary	81	33.1
OND/NCE	19	7.8
Tertiary	11	4.5
**Parity**		
0-3 children	120	49.0
>3 children	125	51.0
Mean ± SD		4.1 ± 2.6
**Preferred number of children**		
≤2 children	81	33.1
2-4 children	81	33.1
>4 children	83	33.9
**Monthly income (naira)**		
None	205	83.7
1,000 - 10,000	12	4.8
11, 000 - 50,000	23	9.4
60,000 - 100,000	5	2.0

**Factors influencing choice of family planning methods:** as revealed in Table 2, the most influential factor identified is that the choice of family planning method was made by the husband. Over half (58.4%) of the women agreed that their husbands chose the method of family planning they ever used for them. One hundred and forty six women (59.6%) confirmed that modern family planning services were readily available. Similarly, one hundred and twenty (49.0%) participants agreed that there were family planning clinics where they could get their families planned while most of them (73.5%) submitted that there were undesirable effects that the family planning had that discouraged them from subsequent use. With regards to the undesirable effects of family planning methods, one of the women in Sasa gave the following explanation: “*I think they use the injectables more but I discovered that the injectables has some undesirable effects like amenorrhea or prolonged menstruation which prevents women from praying or worshiping God. For instance in my own case, when I take the injection, I may not menstruate for that month but when the menstruation comes, it could come for more than two weeks. This hinders a lot of religious exercises. Some people may not menstruate but have varicose veins and so on. We were however, encouraged to go for examination in order to know the best method for everybody. I know some women who decided to go to UCH for the examination but they were told to bring their husbands in order to enable them to be attended to. Some women that didn´t want their husbands to know that they are using family planning couldn´t go back so we had to organize some young men in the community to act as their husbands and follow them to UCH and this worked. These complaints have reduced though*” (IDI, female, politician, Sasa). A greater portion of the participants (57.6%) said their families were not in support family planning. To further explain this, one of the women leaders in Sasa community said that: “*most Hausa men do not like it. So we do not want to give room for problems in anybody's home as this will defeat the aim of the association. The issue of family planning can even cause divorce. As a matter of fact, some men do not even want their women to be in any association, so we need to be careful with whatever we discuss in our meetings*” (IDI, women leader, Sasa).

**Table 2:** factors influencing women's choice of family planning method

Variable	Frequency	Percentage
**My husband chose the method for (N=231)**		
Agree	143	58.4
Undecided	31	12.7
Disagree	57	23.3
**This is the type of family planning method that is most commonly used around here**		
Agree	141	57.6
Undecided	46	18.8
Disagree	44	18.0
**It is the type of family planning method that my religion permits**		
Agree	79	32.2
Undecided	53	21.6
Disagree	99	40.4
**My family likes the kind of family planning method I am using**		
Agree	101	41.2
Undecided	58	23.7
Disagree	72	29.4
**I do not have enough knowledge about the family planning methods available**		
Agree	47	19.2
Undecided	81	33.1
Disagree	103	42.0
**Family planning is expensive**		
Agree	15	6.12
Undecided	68	27.8
Disagree	148	60.4
**It is not readily available**		
Agree	47	19.2
Undecided	38	15.5
Disagree	146	59.6
**There are no family planning clinics close by where I can get my family planned**		
Agree	75	30.6
Undecided	36	14.7
Disagree	120	49.0
**The side effects I experienced or heard about are a source of discouragement**		
Agree	180	73.5
Undecided	18	7.3
Disagree	33	13.5

**Factors influencing family size:** two hundred and eighteen of the participants (89.3%) agreed that since God gives children, their number should not be restricted. To further buttress this point, one of the interviewees stated thus: “*....it is God who gives children and it is whatever number of children God puts in me that I will give birth to, even if it is one million. The white man has no right to tell me to reduce the number of children I have because they are not the ones who are feeding us, neither does the government*” (IDI, male, lawyer, Sasa). One hundred and ninety-five (76.5%) of the participants agreed that having a lot of children will help secure their future. In agreement with this, one interviewee stated that: “*...among them, I would have those that would engage in various professions that would take care of me when I am old*” (IDI, security officer, Sasa). Most (81.6%) participants agreed that their number of children was determined by their husband´s decision. Eighty nine (36.3%) of the participants agreed that they needed children to help them on the farm. One hundred and twenty four (50.6%) of the participants agreed that their cultural beliefs and practices influenced the number of children they have. To emphasise this, one interviewee commented thus: “*Hausa men usually do not marry only one wife and all the wives must bear children. take for instance a man that has four wives, in each year at least two wives must give birth to singletons or multiple children so that is what makes the number of children to be large. Polygamy is a norm in Hausaland*” (IDI, male, security officer, Sasa). More than half of the participants (57.1%) agreed that their religion prescribes the number of children they should have. In agreement with this point, one interviewee stated thus: “*the typical Hausa man wants a lot of children because that is what our religion demands*” (IDI, female, politician, Sasa). Two hundred and four (83.3%) of them agreed that they would have more children because they are still young.

**Figure 3:**
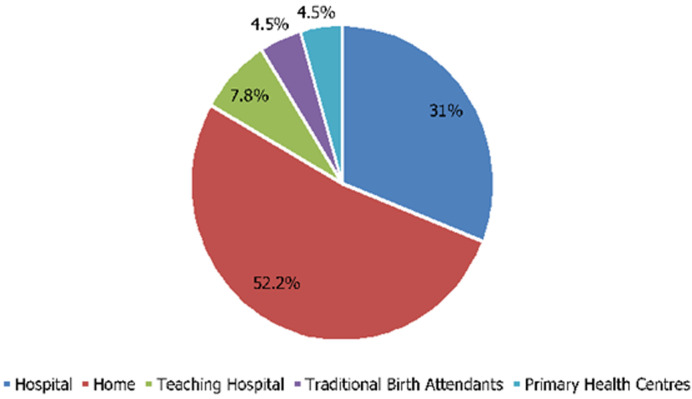
preferred places of delivery

**Factors influencing choice of delivery place:** as revealed in Table 3 and Figure 3, almost half of the population (46.5%) delivered their children at home unattended by skilled personnel. In support of this, one of the women stated that: “*out of my 8 children, only two were delivered in the hospital. The rest were delivered at home*” (IDI, women leader, Sasa). About half of the population (52.2%) preferred home delivery. To further emphasize this, the following comment was captured from the qualitative analysis: “*I prefer delivery at home, especially when it comes with ease and without complications. My wife also prefers to deliver at home*” (IDI, male, transporter, Sasa). Their preferences were based on the following reasons: two hundred and twenty four (91.4%) participants agreed that their husbands chose their delivery places for them. One hundred and sixty five of the participants (67.3%) agreed that their culture did not allow women to go to the hospital for delivery. The influence of culture on the choice of delivery place was further buttressed by one of the interviewees who explained that: “*you know that our culture does not accommodate women who are too fragile or lazy. Women who deliver in the hospital are seen as fragile or weak*” (IDI, women leader, Sasa). Two hundred and one (82.0%) of them agreed that they had people around that assisted at home in order to enable them attend antenatal care. One hundred and seventy one (69.8%) also agreed that they have community health centres available for deliveries. One hundred and twenty-seven (51.8%) of the participants opined that delivery in the hospital is expensive. One hundred and fourteen participants (46.5%) agreed that their religion was against hospital delivery. One hundred and forty (57.1%) of them did not want to be attended to by unfamiliar people. One hundred and seventy two (70.2%) did not want the hospital personnel to impose their policies on them where they would be delivered by a man. This was supported by a response from an interviewee which stated that: “*the problem I have with delivering in the hospital is when I always have to lie down, confined to one place. This will increase the pain. At home, I have the freedom to move about and work till the baby is out. Most times I give birth on my knees which will not be allowed in the hospital*” (IDI, women leader, Sasa).

**Table 3:** factors influencing the women's choice of delivery

Variable	Frequency	Percentage
**Places of delivery used by the women**		
At home by a skilled professional	34	13.9
At home by an unskilled attendant	114	46.5
Traditional birth attendant	9	3.7
Primary health care centre	30	12.2
Teaching hospital	58	23.7
**Preferred place of delivery**		
Hospital	76	31.0
Home	128	52.2
Teaching hospital	19	7.8
Traditional birth attendants	11	4.5
Primary health centres	11	4.5
**Determinant factors**		
**My husband's decision**		
Agree	224	91.4
Undecided	1	0.4
Disagree	20	8.2
**My culture does not allow people to go to the hospital**		
Agree	165	67.3
Undecided	18	7.3
Disagree	62	25.3
**My mother in-law assists me with the house chores so that I can attend antenatal clinics and other necessary clinical check-ups**		
Agree	201	82.0
Undecided	27	11.0
Disagree	17	6.9
**I have enough knowledge about the delivery places**		
Agree	185	75.5
Undecided	44	18.0
Disagree	16	6.5
**Community health centres are available for deliveries**		
Agree	171	69.8
Undecided	48	19.6
Disagree	26	10.6
**I have experienced the various options and decided to choose this one**		
Agree	127	51.8
Undecided	67	27.3
Disagree	51	20.8
**Delivery in the hospital is expensive**		
Agree	127	51.8
Undecided	37	15.1
Disagree	81	33.1
**My religion is against delivery in the hospital**		
Agree	114	46.5
Undecided	27	11.0
Disagree	104	42.4
**I do not want to be attended to by unfamiliar people**		
Agree	140	57.1
Undecided	27	11.0
Disagree	78	31.8
**I do not want the hospital personnel to impose their hospital policies on me, where I may be delivered by a man**		
Agree	172	70.2
Undecided	8	3.3
Disagree	65	26.5

## Discussion

This study revealed that a large portion of the population (93.7%) was aware of modern family planning methods. This is in line with National Demographic Health Survey´s 2008 which states that 72% of all women and 90% of all men know at least one method of contraception. The level of awareness however, contradicts the findings from a study that was conducted by Wall (1998) that few Hausa women have any knowledge about birth control. This increased level of awareness may be attributed to the technological advancement and the improved awareness on the mass media.

From the study, the major source of information about family planning was television (Figure 2). This finding is at variance with the findings of the study conducted by Omolase, Faturoti and Omolase in 2009 among the Yoruba women that revealed that the major source of information about family planning was healthcare workers. This may be due to the fact that most of the Yoruba respondents sought healthcare at the healthcare institutions making accessibility of information about family planning from healthcare workers easier. The role of mass media in disseminating such vital information cannot be overemphasized. This is much easier because information can reach a large number of people at the same time [12]. However, a lesser proportion (43.3%) of the study participants used these family planning methods, indicating the need to translate the high awareness of family planning to use which was also stated by Umar and Mohammed in 2014. This finding also confirms the study which was conducted by Nettey *et al*.in 2014 and the report from NDHS in 2014 which stated that awareness of family planning was more than its use [10,13]; despite the fact that most of them accepted that there are family planning clinics where they could easily access family planning services at an affordable rate [14].

Poor use was attributed to some influencing factors such as the husband´s perception, educational status, marital status, income, cultural and religious beliefs. This finding reiterates the findings of a research that was conducted by Eyayou, Berhane and Zerihun in 2004 among Kanuri women of Borno state which revealed that very few of the women used modern family planning methods because majority of their husbands disapproved of it. Other reason for non-use of family planning was that God who gave the children will surely provide for them [15]. Some of the women also stated that they were not using family planning because they were still young and desired to have more children. Experiencing undesirable effects of contraceptives such as injectables was also implicated [16].

Religion and cultural beliefs are factors mostly identified to influence family size. Some of the participants explained during the in-depth interview that their religion (Islam) encourages them to have as many children as possible. This makes them reluctant to accept family planning because they feel they would not be doing the will of God if they restrict their number of children. Studies have shown that worldwide, Muslims have the highest fertility rate, an average of 3.1 children per woman, due to young age at marriage compared to other religious groups [17]. Another factor influencing family size may likely be the level of education. A predominant number of the women who participated in this study had either no formal education or primary education (Table 1). In a study conducted in Ondo state, it was discovered that the educated people had fewer children than the uneducated; the researchers´ observation revealed that illiterates seem to keep on procreating ceaselessly without having the resources to cater for them [18].

Orbeta (2005) discovered a striking positive relationship between family size and poverty incidence and severity. He also showed how a large family size creates the conditions leading to greater poverty through its negative impact on household savings, labour force participation and earnings of parents, as well as the human capital investment in children [19]. Similarly, Renjhen, Kumar, Pattanshetty, Sagir and Samarasinghe (2010) stated that uncontrolled population growth is recognized as the single most important impediment to national development [20]. Therefore, the promotion of family planning, especially in countries with high birth rates like Nigeria, has the potential of reducing poverty and hunger and averts 32% of all maternal deaths and nearly 10% of childhood deaths. It may also substantially contribute to women´s empowerment, achievement of universal primary schooling and long-term environmental sustainability [21].

Most of the participants (46.5%) delivered their children at home, unattended to by skilled personnel and preferred home delivery. This is in support of the results from the NDHS (2008), revealing that 62% of births occur at home. This preference could be influenced by various factors including their cultural beliefs which make them feel that delivery in the hospital makes them inferior to their peers. Religion has also been implicated as a factor that influences the women´s choice of delivery place. The institution of the Purdah segregates Muslim women from public places women and this greatly impacts on the health- seeking behaviours [4].

More than half of the participants (51.8%) agreed that delivery in the hospital is expensive and this is congruent with their approximate financial income per month. Majority of the participants are full time housewives who earn close to nothing in a month (Table 1). A good number of women (82.0%) agreed that they have people to take care of the household while they go for antenatal and other healthcare services. This, however, did not encourage them to deliver in hospital. This finding is in support with that of Gbadamosi (2010) who also found out that in virtually every community, facilities recorded high antenatal care (ANC) attendance, but recorded abysmal delivery figures [22].

Other factors such as poor awareness about delivery places; high parity which makes them feel highly experienced and capable of taking care of themselves; and their low educational qualification were also identified to have affected the women´s choices of delivery place.

## Conclusion

Although the level of awareness about family planning and contraceptive methods is quite satisfactory, the level of contraceptive uptake is not optimal among the study population. In this regard, more adapted educational and counselling interventions should be undertaken among women, with the inclusion of family planning messages directed at men too. This is necessary because men inclusion has been reported to impact women´s uptake of maternal health services. Hausa men should be also educated on the likely implications and consequences of making unhealthy maternal choices.

Most Hausa women prefer to give birth at home. This preference could predispose them to complications such as severe bleeding leading to shock, excruciating pain, vesico-vaginal fistula, and spread of infections like HIV and hepatitis and painful intercourse later, burn injuries, severe hypertension, eclampsia and heart failure and subsequent death which are associated with harmful practices that occur during home delivery. There is need for education and encouragement of the women to seek adequate and appropriate healthcare services in order to reduce the rate of maternal and child mortality.

Religion has also been implicated as a factor that influences the women´s choice of delivery place. The institution of the Purdah segregates muslim women from public places and greatly impacts on their health-seeking behaviours. In view of this, the women could be encouraged to get a family doctor or nurse who could attend to their health during delivery. Home deliveries in such communities should be structured and planned where birth preparedness and complication readiness principles are applied. The religious leaders should be educated on the relevance of making healthy decisions.

### What is known about this topic

- In developing nations like Nigeria, children are valued as they not only demonstrate the masculinity of the men but equally provide the extra useful hands on the farms in communities such as the Hausa settlements where agriculture is the major source of income. Besides, aged parents and extended family relations depend on their children for maintenance at old age [5]. Hence, they are reluctant to limit the number of children they give birth to;
- Diverse socio-cultural beliefs are in line with strong preference for polygamy and these beliefs hold that children are a blessing from God and insurance against old age. The fact that a man has the final say in the family has also to an extent determined what, when and probably how a family planning method would be carried out [3]. The HAUSA community is patrilineal with a strong male influence on many decisions concerning the household including reproduction. This makes the attitude of the male counterpart towards family planning and the number of children to be born an important factor influencing fertility level among the Hausas;
- Level of awareness about family planning methods among Hausa women studied in Ibadan is relatively high but there is low use of modern family planning methods among Hausa women in Ibadan.

### What this study adds

- Reasons such as religious beliefs, culture, spouses´ disapproval and delayed fertility following use, were identified as barriers to the use of modern family planning methods among Hausa women in Ibadan;
- The study revealed that many Hausa women preferred large family size and home delivery without interventions of skilled personnel. Factors responsible for these include religious and cultural inclinations, values and beliefs; inadequate finances, husband´s choice and personal/significant others´ myths and misconceptions;
- This study adopted both quantitative and qualitative methods to explore the reasons for maternal health choices and provided insights on viable nursing interventions that can be adopted promote skilled birth delivery to reduce maternal morbidity and mortality.
